# How parents and children evaluate emollients for childhood eczema: a qualitative study

**DOI:** 10.3399/BJGP.2021.0630

**Published:** 2022-05-24

**Authors:** Eileen Sutton, Alison RG Shaw, Matthew J Ridd, Miriam Santer, Amanda Roberts, Helen Baxter, Hywel C Williams, Jonathan Banks

**Affiliations:** Bristol Population Health Sciences Institute, University of Bristol, Bristol.; Bristol Population Health Sciences Institute, University of Bristol, Bristol.; Bristol Population Health Sciences Institute, University of Bristol, Bristol.; Primary Care Research Centre, University of Southampton, Southampton.; Nottingham Support Group for Parents of Children with Eczema, Nottingham.; Bristol Population Health Sciences Institute, University of Bristol, Bristol.; Centre of Evidence-Based Dermatology, University of Nottingham, Nottingham.; Population Health Sciences, Bristol Medical School, University of Bristol, Bristol.

**Keywords:** eczema, emollients, dermatitis, atopic, ointments, pediatrics, primary health care

## Abstract

**Background:**

Eczema affects one in five children in the UK. Regular application of emollients is routinely recommended for children with eczema. There are four main emollient types, but no clear evidence of which is best. The current ‘trial and error’ approach to find suitable emollients can be frustrating for parents, children, and clinicians.

**Aim:**

To identify how parents and children experience and evaluate emollients.

**Design and setting:**

Qualitative interview study, nested within a primary care trial of emollients (Best Emollients for Eczema [BEE] trial).

**Method:**

Semi-structured interviews with children with eczema and their parents were conducted. Participants were purposively sampled on emollient type (lotion, cream, gel, or ointment), age, and eczema severity.

**Results:**

Forty-four parents were interviewed, with children participating in 24 of those interviews. There was no clear preference for any one emollient type. The strongest theme was the variation of experience in each of the four types. Participants focused on thickness and absorbency, both positively and negatively, to frame their evaluations. Effectiveness and acceptability were both considered when evaluating an emollient but effectiveness was the primary driver for continued use. For some, participating in the trial had changed their knowledge and behaviour of emollients, resulting in use that was more regular and for a longer duration.

**Conclusion:**

There is no one emollient that is suitable for everyone, and parents/children prioritise different aspects of emollients. Future research could evaluate decision aids and/or tester pots of different types, which could enable clinicians and parents/children to work collaboratively to identify the best emollient for them.

## INTRODUCTION

Childhood eczema (also known as atopic eczema/atopic dermatitis) is a common long-term condition affecting around one in five children in the UK.^1^ Symptoms of itching and pain, owing to dry and inflamed skin, can lead to emotional, psychological, and quality-of-life issues for children and families.^2^^–^^5^ Most children are managed in primary care, and because there is no cure the focus is on disease control using topical treatments. Consequently, eczema requires a high level of self-management, with anti-inflammatories such as topical corticosteroids for skin inflammation and regular moisturisation with emollients for maintenance and prevention of flares.^6^^,^^7^

Emollients or moisturisers work by adding water to the skin, reducing water loss, and acting as a barrier to external irritants that can inflame the skin.^8^ There are four main types: lotions (thin) through creams and gels to ointments (thick), usually applied twice daily or more frequently. Numerous emollients are available, on prescription or over the counter, and there is no clear evidence that any one is better than another.^9^ As a result, children, families, and primary care clinicians tend to adopt a trial-and-error approach until they find an emollient that works.^10^^–^^13^ Such an approach puts a strain on families and adds to the pressure on primary care resources.^6^^,^^7^

Guidelines, systematic reviews, and research prioritisation exercises have recommended research into eczema treatment and management to identify which emollients are effective and safe.^9^^,^^14^^,^^15^ The Best Emollients for Eczema (BEE) randomised controlled trial compared creams, lotions, gels, and ointments in children aged 6 months to 11 years.^16^ The BEE trial incorporated a nested qualitative study, which is reported here. The aim was to identify parents’ and children’s experiences of emollient use, and how they evaluated the effectiveness and acceptability of the four emollient types.

## METHOD

This was a qualitative interview study with parents of children with eczema participating in the BEE study. Participants in the trial were randomised to use a lotion, cream, gel, or ointment as their only leave-on emollient for 16 weeks (the primary outcome period). The interviews were conducted at approximately 4 weeks and 16 weeks after randomisation (see Box 1 for information about the BEE trial). Participating children were invited to join the interviews.

**Box 1.** Background information on the Best Emollients for Eczema trial

Participants	Children with eczema aged 6 months to 11 years
Intervention	Randomised to use a lotion, cream, gel, or ointment as their only leave-on emollient for 16 weeks
Primary outcome	Patient-Orientated Eczema Measure over 16 weeks
Follow-up	Weekly questionnaires for Weeks 1–16; four weekly questionnaires thereafter. Participants could continue with their allocated emollient or switch to an alternative between Week 16 and Week 52.

All interviews explored experiences of using the different emollients. Week 4 interviews looked at initial acceptability, the extent to which emollient use was consistent with recommended use, and intentions regarding ongoing use. In addition, interviews at Week 16 covered the overall experience and which type of emollient(s) participants intended to use going forward.

**Table:** How this fits in

There is limited evidence on how children with eczema and their parents use and evaluate emollients, commonly referred to as ‘moisturising creams’. Among parents and children using different types of emollients, wide variability of experiences within and across different emollients was found. There is no one emollient that is suitable for everyone, and families would welcome support in finding emollients that work for them on different areas of the body and at different levels of eczema severity. Future research should focus on developing tools and resources that will support clinicians and families from all backgrounds to identify the best emollient for their children.

### Sampling

Children were purposively sampled by: emollient type; recruiting centre (Bristol, Southampton, or Nottingham); age (0.5–<7 years/≥7–11 years); eczema severity (mild, moderate, or severe, determined by Patient-Oriented Eczema Measure at baseline);^17^ and cessation of allocated emollient use. Week 16 interviewees were also sampled on future intentions regarding their allocated emollient, as captured by the trial Week 16 questionnaire. The experience of emollient use by children and parents from different ethnic backgrounds was not one of the original research questions for the qualitative study or the BEE trial. However, following discussions within the BEE trial management group, a decision was made to try to include parents whose children came from a range of different ethnicities, to capture experiences of emollient use for different skin types.

### Recruitment

All parents received information about the qualitative study at the time of recruitment into the BEE trial and were asked for consent to be contacted for interview. Potential interviewees were invited by email and provided with detailed information. Parents who agreed to participate gave informed written consent and participating children gave written assent. Recruitment stopped when there was agreement that inductive thematic saturation had been reached.^18^

### Data collection

Interviews were conducted face-to-face or by phone and supported by the use of topic guides (see Supplementary Appendix S1). These were shaped by the study aims, a feasibility study,^10^ and the wider literature. There were separate topic guides for parents and children. All interviews were audiorecorded.

### Data analysis

Interviews were transcribed verbatim by a professional transcriber and anonymised. Coding and data management were supported by NVivo (version 12) software.

Analysis took place alongside data collection in an iterative process, to allow insights from earlier interviews to shape future interviews.^19^

Using a thematic approach,^20^ analysis incorporated a combination of deductive (based on the study aim) and inductive (further engagement with data) coding strategies to develop a preliminary coding framework. A research subgroup read and independently coded a subset of interview transcripts and met regularly to review analysis and refine the coding frame. The team also incorporated feedback from the Patient and Public Involvement panel and the Trial Management Group. Once finalised, the coding framework was applied to the dataset.

Following coding, data were examined and compared both across and within trial groups. Themes and subthemes were identified and refined through continual comparison of data elements with each other in an iterative manner. A narrative summary of the findings from the interviews was produced, attending to areas of divergence and convergence in the datasets and the different perspectives represented. This formed the basis of the results presented below.

## RESULTS

Forty-four interviews with parents were undertaken between 21 February 2018 and 17 September 2019: 20 at Week 4 and 24 at Week 16. These included five parents, interviewed twice at Week 4 and Week 16, who had expressed an interest in undertaking follow-up interviews and whose children’s characteristics aligned with the sampling criteria. The characteristics of the children are shown in Table 1. Twenty-four children participated in the interviews with their parents. Interviews lasted between 15 and 61 minutes (mean = 28 minutes). Eighteen interviews were face-to-face and 26 were by phone. Only 10 parents of children from non-White backgrounds were recruited, and as such it was not possible to fully explore the experiences of people with a range of skin types. This reflected the number (*n* = 77/550, 14%) of non-White participants recruited to the wider BEE trial.

**Table 1.** Characteristics of children sampled (interview participants were primarily parents/carers)

	**Week 4**				**Week 16**				

	**Lotion**	**Cream**	**Gel**	**Ointment**	**Lotion**	**Cream**	**Gel**	**Ointment**	**Total**
**Participants**	**5**	**5**	**5**	**5**	**6**	**6**	**7**	**5**	**44**

**Children**	**3**	**4**	**4**	**1**	**3**	**3**	**3**	**3**	**24**

**Eczema severity**									
Mild	1	2	1	2	2	3	2	1	14
Moderate	3	3	3	2	2	2	4	3	22
Severe	1	0	1	1	2	1	1	1	8

**Age, years**									
0.5–<7	2	2	1	4	3	2	3	2	19
≥7–11	3	3	4	1	3	4	4	3	25

**Emollient use status**									
Stopped before primary outcome (Week 16)	1	1	1	3	2	3^a^	0	0	11
Intending to carry on	—	—	—	—	4	3	4	3	14
Change after primary outcome (Week 16)	—	—	—	—	0	0	3	2	5

**Ethnicity**									
White British/White/White Other	5	5	2	4	3	6	5	4	34
Black/African/Caribbean/Black British	0	0	0	1	0	0	0	0	1
Asian	0	0	1	0	1	1	0	0	3
Mixed/multiple ethnic groups	0	0	2	0	2	0	1	1	6

**Recruiting centre**									
Bristol	3	2	2	3	3	1	2	3	19
Nottingham	1	1	1	1	2	2	2	1	11
Southampton	1	2	2	1	1	3	3	1	14

*Numbers in each cell represent interviews conducted for each of the sampling criteria. 5 of the 16-week interviews were with families who had been interviewed at Week 4. Recruitment at Week 4: 27 invited; 20 completed; 1 booked but unable to contact; and 6 did not reply. Recruitment at Week 16: 41 invited; 24 completed; 1 booked but unable to contact; 1 booked but cancelled owing to staff illness; 2 refused; 1 too busy; and 12 did not reply.*

a *Family stopped using study emollient at Week 4 and was subsequently interviewed at Week 16 and also recorded as ‘stopped’ at second interview.*

The findings are reported under three themes directly related to the study aims:

• effectiveness and acceptability (how well the emollients worked for participants and how they found using and integrating them into everyday life);

• balancing choice between effectiveness and acceptability (the value placed on different aspects of the emollients and how this shaped their decisions on future emollient use); and

• study participation and the use of emollients (how study engagement and supporting materials affected emollient application routines and practices).

Quotes are tagged by ‘study ID’ and ‘child’ if drawn from children’s data, eczema severity, age of child, and assigned emollient.

While ‘cream’ is a type of emollient, the term ‘cream’ or ‘moisturising cream’ was also used by parents/carers and children to describe emollients and moisturisers generically. When used in this way, the term [generic] has been added in the data after ‘cream’. Where ‘cream’ appears without [generic], this references ‘cream’ as an emollient type.

### Emollient effectiveness and acceptability

#### Emollient effectiveness

A clear theme identified in relation to effectiveness was the variation of experience within each emollient type. All four types were associated with both positive (stabilisation or improvement) and negative (adverse reactions or worsening) experiences.

Participants identified their experience of an emollient’s effectiveness in positive terms if it stabilised and/or improved their child’s eczema.

For the former, the key aspect was that the emollient reduced or stopped disease flares, and in doing so reduced the need for parents to use a topical corticosteroid:

*‘* [The emollient] *doesn’t always necessarily get rid of the eczema but sometimes it just stops it getting any worse, stops her getting any flare ups.’*

(N6, mild, aged 6 months, lotion)

For some parents the effect of their study emollient was more beneficial, leading to an improvement in their child’s signs and symptoms:

*‘We’ve tried a few different ones and ‘cos we’d got used to* [a lotion] *, I was a bit worried about changing it but it changed for the positive actually.’*

(S7, moderate, aged 7 years, cream)

*‘We currently use* [the study ointment] *and we have used it and since then it has cleared up beautifully, like round her neck and on her arms you can barely tell she’s* [got eczema].’

(B11, moderate, aged 8 years, ointment)

However, there were negative experiences across all emollient types. These ranged from skin irritation to worsening of eczema and flares, which often led to parents changing to an alternative or reverting to their pre-study emollient:

*‘By the end of that day it was red and really bad, and within a couple of days she was screaming and crying in pain whenever we were putting* [the gel] *on her … so we were probably only on it three or four days I think.’*

(B7, severe, aged 3 years, gel)

*‘I think within two or three weeks his skin was worse than it had ever been … the* [cream] *obviously wasn’t good enough to then control it and it went nuts … ’*

(S8, mild, aged 1 year, cream)

#### Effectiveness and emollient thickness

In describing why an emollient did or did not work for them, a common theme was the thickness of an emollient, which was linked to its moisturising and protective qualities:

*‘I think it’s better than some of the things we’ve used in the past ‘cos of its consistency and it has kept the dryness at bay.’*

(S5, moderate, aged 4 years, cream)

*‘* [The lotion] *was so thin and watery, it had no effect whatsoever and we had to go, can’t remember if we went to the doctors and got a different like better emollient ‘cos it just wasn’t effective.’*

(B16, moderate, aged 7 years, lotion)

For others, it was the ability of a thinner emollient to absorb into the skin that was seen as the effective attribute. The following participant stopped using their study ointment and reverted to thinner emollients they had previously used:

*‘I like it to soak in. I think when you’ve got one of these gels, like on the skin’s really greasy, I don’t think it’s nice for anybody. So I think, like I say, that it soaks in and then obviously that it has an effect.’*

(N5, moderate, aged 2 years, ointment)

#### Acceptability, absorption, and emollient thickness

Participants’ accounts of acceptability also varied within and across the emollient types, with no one being clearly more or less acceptable than another. It was also evident that descriptions of what made an emollient acceptable varied between participants.

As with effectiveness, key factors were thickness and absorbency. Some prioritised an emollient that absorbed into the skin quickly, was not sticky, and did not mark clothing, whereas others expressed a need for an emollient that sat more prominently on the skin and provided a ‘protective barrier’; for example, to enable participation in swimming.

For some participants the thinner emollients (lotions) and gels were associated with ease of application. The thinner nature of these emollients meant that they would be absorbed into the skin quicker and not have a negative impact on clothing:

*‘I think just the ease of the emollient, you know, being absorbed by the skin is quite helpful because when it’s … just sitting on top is not something very attractive to use.’*

(S2, moderate, aged 11 years, gel)

These same qualities were also associated with negative attributes, with some participants feeling the thinner consistency does not give lasting ‘protection’ and the lack of visible presence of the emollient resulting in a perception that more is needed to provide protection, thereby increasing the time spent applying the emollient:

*‘I think the lotion that he’s got now soaks in a lot better. Obviously it’s a lot thinner so … I’ll put some on, just a little bit, rub it in and almost make sure it’s soaked in and then I tend to put some more on whereas with the thicker one you could put one lot on, you could kind of see it all and know it was going to stay on.’*

(B4, mild, aged 8 years, lotion)

The thicker emollients tended to be described as more challenging to use and as having a greater impact on everyday life, particularly the time spent bathing and dressing:

*‘* [The ointment] *does absorb into the skin though not as quickly and I found it was particularly difficult because … it was adding extra time … I had to get* [my son] *up early ‘cos the cream it sunk in, you turn around and its sunk in, the ointment takes what felt like quite a lot longer so I almost had to put a towel, do him head to toe, let him lie on the towel while I was getting … baby ready or whatever and then come back, check, and then if we were putting steroid on then we’ve got to apply the steroids and then go off again and then ok, right, well you can get dressed now.’*

(B17, moderate, aged 5 years, ointment)

*‘It’s pretty hard to rub in and it takes like three or four minutes to rub in usually.’*

(B10, [child] moderate, aged 11 years, cream)

Thickness was also identified as a positive aspect in applying an emollient:

*‘It’s better than the old one cos it’s spreadable.’*

(B13 [child], mild, aged 8 years, ointment)

Conversely, thicker emollients caused some users discomfort:

*‘It’s like clay. I don’t really like the feeling and like what it does to my skin. It kind of makes my skin sticky and then it feels weird, when I put it on my skin, inside my skin it feels like really weird.’*

(S4 [child], moderate, aged 8 years, gel)

*‘I think the one that we got given for our test is pretty awful, it’s like Vaseline.’*

(S9, [child], severe, aged 7 years, ointment)

#### Emollient containers and dispensers

Acceptability also related to the type of container the study emollient was dispensed from. Containers varied across different emollient types and included pumps, squeezable bottles, tubs, and tubes. Overall, participants were positive about pumps:

*‘It’s just so easy. You just leave it on the surface, quick pump and then you’re kind of done … when you’ve got two children with eczema and you’ve got to get everybody ready for bed or ready for school in the morning that kind of thing does make a difference ‘cos it’s just quicker.’*

(B14, moderate, aged 8 years, gel)

Participants expressed negative views about tubs, in which all ointments come, and which require a spoon to scoop out the required amount. Participants described this as time consuming, and some used their hands instead (which increases the risk of infection):

*‘When I went to see the dermatologist she said that the best way, or what the advice should be that you scoop it out with a spoon … I did use my hands … but I think that adding a spoon to that just … its adding some, you know, it’s another something to do isn’t it, something else to clean up, whereas that pump is so* [easy].’

(B16, moderate, aged 7 years, lotion)

### Emollient choice: balancing effectiveness and acceptability

This section looks at the choices that families made around emollient use, with a particular focus on intentions after Week 16, when participants could choose to continue with their study emollient or switch to an alternative. In making their choices, participants considered effectiveness and acceptability in assessing an emollient, not necessarily in opposition to each other:

*‘It was quite good. It helped* [with itching] *… but it is quite greasy.’*

(S9 [child], severe, aged 7 years, ointment)

For some participants there was a clear improvement, and it was therefore an easy decision for them to carry on using their allocated emollient:

*‘The fact that it seems to be helping* [was a] *good incentive, so we weren’t counting down the weeks until the end of the study, having to decide whether or not it was worth carrying on … there was nothing really to think about.’*

(B6, moderate, aged 10 years, lotion)

The data showed effectiveness was the primary driver of decision making. This was evident in cases where parents recognised the improved acceptability of their assigned emollient but were unable to keep using it because it did not control eczema in the way that their previous emollient had done:

*‘It went on really nicely and she was quite excited about having this new cream* [generic] *… It just didn’t solve, yeah, if anything it made it so worse. I think if it had just kept it the same, I would have probably carried on using it but I felt it was making it worse and so couldn’t then carry on.’*

(N5, moderate, aged 2 years, ointment)

*‘Because it’s so thick and it’s hard to get out ‘cos of how much you get out and it just like stings my body when I put it on … I would have stayed with it because it’s making me better but … it’s not.’*

(B10 [child], moderate, aged 11 years, cream)

The data also highlighted the value placed on acceptability by participants, especially when the effectiveness of the emollient was comparable with emollients they had previously used:

*‘Actually, there is improvement, not necessarily in his skin but I think like* [my son] *was saying about it’s easier to apply, I think that’s where the improvement is. It’s easier to apply, it’s not as sticky … and uncomfortable as the other one.*’

(B13, mild, aged 8 years, ointment)

There were also instances where participants managed choice going forward by using different emollients for different parts of the body, prioritising effectiveness in badly affected areas and using an emollient that was easier to apply in other areas:

*‘We carried on using* [the gel] *because on the skin that wasn’t broken it was fine and it seems to do a decent enough job. I would say it’s just as good as the [lotion] that we’ve been using. It seems to give the same kind of level of moisturising and it’s kinder to his clothes, it’s not really greasy, it seems to soak in well. So we carried on using it and then just literally would put something else on his face and hands when he needed it.’*

(B18, mild, aged 4 years, gel)

### Study participation and the use of emollients

As part of the trial, participants were provided with an Emollient Information Sheet about how emollients should be applied. Some parents reported, particularly in the Week 16 interviews, that engaging with the study information had improved their knowledge of eczema management and emollient types, and changed their use of emollients:

*‘I’ve only ever had that from the BEE study actually, advice about baths and things like that, about how to put on* [emollients]. *That’s the first time I’d ever heard of that.’*

(S7, moderate, aged 7 years, cream)

As part of the trial, participants were advised to use their study emollient at least twice daily, and the interviews explored whether their daily routines differed to their pre-study practice. Some participants commented that more regular application may have contributed to an improvement in their child’s skin, as before participation they could sometimes be ‘slack’ or ‘lazy’:

*‘We probably haven’t been as diligent in terms of applying it on a daily basis as we have been with the study … because we said we would, we’ve probably only used the* [pre-study emollient] *when we’ve needed to, when he’s had really dry skin.’*

(N4, moderate, aged 8 years, gel)

The trial also asked participants to answer questions on a weekly basis about eczema symptoms and treatment use during the first 16 weeks. For some participants, engaging with the study surveys acted as a reminder to apply their emollient regularly, rather than using it to manage an exacerbation of eczema. Participating in the trial had changed some parents’ approach to how long they were willing to persist with an emollient. Committing to an emollient for a sustained period of 16 weeks led some to see benefits in using regularly over a longer period:

*‘I don’t think we ever gave it long enough in terms of, you know, we’d get this* [emollient] *and then we’d go back to the doctors and they’d say oh it’s not really working so change it and I think in hindsight … and also based on this experiment that he’s done with the cream* [generic] *… we would never have given* [an emollient] *this long to work.’*

(S10, severe, aged 6 years, lotion)

## DISCUSSION

### Summary

This study explored parents’ and children’s use of emollients through the lenses of perceived effectiveness and acceptability. The findings show that no emollient types were identified by the interviewees as either more effective or more acceptable than any other, and participants’ responses varied across all four emollient types. Effectiveness was prioritised over acceptability in shaping decisions about emollient use, although acceptability was also important and came to the fore when effectiveness was on a similar level as previously used emollients. In terms of how participants evaluated emollients, thickness and absorbency were key considerations. Thicker emollients were liked because of their ‘protective’ and long-lasting qualities, but tended to affect clothing and were difficult to apply. The lighter emollients absorbed into the skin, which for some highlighted moisturising capability but for others indicated a lack of ‘protection.’ Participants highlighted how containers effected acceptability, with pumps being preferred over tubs. Finally, participating in the trial changed perceptions for some participants about how emollients should be used, both in terms of their awareness of different emollient types and in their willingness to persist with using emollients over a prolonged period.

### Strengths and limitations

To the authors’ knowledge, this is the first qualitative study exploring the four main types of emollients. It sampled across a range of participant characteristics from all four trial groups. However, it was not possible to recruit sufficient numbers from Black and ethnic minority families to comment on using different emollients with different skin types.

The study endeavoured to capture the views of children, and the direct input of those experiencing the emollients is a strength.

Data were collected face-to-face and by telephone interviews, which can effect depth of data.^21^ No differences were found between interview modes, but phone interviews including children were more challenging, which may have affected their contribution.

### Comparison with existing literature

There is a body of qualitative childhood eczema research^22^ focused on the lived experience of eczema for children and families,^23^^–^^25^ beliefs around eczema and its causes,^26^^,^^27^ and the management and treatment of eczema.^24^^–^^28^ Studies have highlighted confusion about different emollient types and concern about different emollient constituents.^22^ However, only Santer *et al* ^29^ looked at perceptions of emollients among patients or carers. Their study used interview data with carers of children with eczema aged ≥5 years. They identified similar trade-offs around acceptability and effectiveness as were observed in the present study. The present results add to these findings because it assesses experiences across the main four types of emollients in greater depth, and includes the views of children.

There has been limited qualitative work directly with children^30^^–^^32^ and adolescents/young adults with eczema.^33^^–^^35^ Teasdale *et al* ^36^ interviewed 14 children (predominantly girls, with mild to moderate eczema) aged 6–12 years. They found that applying ‘creams’ (in the generic sense) was helpful in relieving eczema symptoms. As in the present interviews, participants had mixed views about the texture, viscosity, and odour of some topical treatments, yet reported using topical treatments even if they disliked its texture or odour, supporting the trade-off favouring effectiveness over acceptability.

### Implications for research and practice

This study shows that no one emollient will suit all children with eczema and supports the offer of a wide choice of emollients for families. Therefore, clinicians should focus on supporting families in finding an emollient or emollients that meet their needs and preferences. This includes asking about when the emollients will be used and by whom (for example, at home with a parent, and/or in school by the child), and their experience and opinions of previous emollients. This information can inform which emollients to try or avoid. In addition, the study materials (including an emollient information sheet and regular surveys) promoted emollient use by some families. Prescriptions should be accompanied with information to support emollient use; for example, an eczema Written Action Plan,^37^ which contains links to videos on how and when to apply emollients.

Future research could look at ways of bringing user views and preferences, including those from Black and ethnic minority groups, into the decision-making process, by providing samples of all four types of emollients in tester pots to enable direct comparison (in the BEE trial participants were only allocated one type to try). Such an approach could be trialled in community pharmacy as well as primary care. An alternative approach could be to develop a patient decision aid,^38^ which summarises the characteristics of emollient types, to see if eliciting prior preferences and providing information on choices improves the experience of finding an emollient that ‘works’ for the patient. This would need to consider cultural sensitivities and communication preferences across different ethnic populations.
